# Cysteine proteinases from papaya (*Carica papaya*) in the treatment of experimental *Trichuris suis* infection in pigs: two randomized controlled trials

**DOI:** 10.1186/1756-3305-7-255

**Published:** 2014-05-30

**Authors:** Bruno Levecke, David J Buttle, Jerzy M Behnke, Ian R Duce, Jozef Vercruysse

**Affiliations:** 1 Department of Virology, Parasitology and Immunology, Faculty of Veterinary Medicine, Ghent University, Salisburylaan 133, Merelbeke, Belgium; 2 Department of Infection & Immunity, University of Sheffield Medical School, Beech Hill Road, Sheffield S10 2RX, UK; 3 School of Life Sciences, University of Nottingham, University Park, Nottingham NG7 2RD, UK

**Keywords:** *Trichuris*, Papaya, Cysteine proteinases, Ethno-medicine, Soil-transmitted helminths, Egg reduction rate, Worm reduction rate

## Abstract

**Background:**

Cysteine proteinases (CPs) from papaya (*Carica papaya*) possess anthelmintic properties against human soil-transmitted helminths (STH, *Ascaris lumbricoides*, *Trichuris trichiura* and hookworm), but there is a lack of supportive and up-to-date efficacy data. We therefore conducted two randomized controlled trials in pigs to assess the efficacy of papaya CPs against experimental infections with *T. suis*.

**Methods:**

First, we assessed efficacy by means of egg (ERR) and adult worm reduction rate (WRR) of a single-oral dose of 450 μmol active CPs (CP450) against low (inoculum of 300 eggs) and high (inoculum of 3,000 eggs) intensity *T. suis* infections and compared the efficacy with those obtained after a single-oral dose of 400 mg albendazole (ALB). In the second trial, we determined and compared the efficacy of a series of CP doses (45 [CP45], 115 [CP115], 225 [CP225], and 450 [CP450] μmol) against high intensity infections.

**Results:**

CP450 was highly efficacious against both levels of infection intensity, resulting in ERR and WRR of more than 97%. For both levels of infection intensity, CP450 was significantly more efficacious compared to ALB by means of WRR (low infection intensity: 99.0% vs. 39.0%; high infection intensity; 97.4% vs. 23.2%). When the efficacy was assessed by ERR, a significant difference was only observed for high intensity infections, CP450 being more efficacious than ALB (98.9% vs. 59.0%). For low infection intensities, there was no significant difference in ERR between CP450 (98.3%) and ALB (64.4%). The efficacy of CPs increased as a function of increasing dose. When determined by ERR, the efficacy ranged from 2.1% for CP45 to 99.2% for CP450. For WRR the results varied from -14.0% to 99.0%, respectively. Pairwise comparison revealed a significant difference in ERR and WRR only between CP45 and CP450, the latter being more efficacious.

**Conclusions:**

A single dose of 450 μmol CPs provided greater efficacy against *T. suis* infections in pigs than a single-oral dose of 400 mg ALB. Although these results highlight the possibility of papaya CPs for controlling human STH, further development is needed in order to obtain and validate an oral formulation for human application.

## Background

The London Declaration on Neglected Tropical Diseases (NTD) brought an international awareness and commitment to combat NTD that has since resulted worldwide in increased pledges of drug donations [1]. However, these worldwide prospects for more frequent mass drug administration (MDA) campaigns warrant caution, particularly for soil-transmitted helminths (STH, *Ascaris lumbricoides, Trichuris trichiura* and hookworms). First, MDA programs to control STH predominantly rely on just one group of anthelminthics, the benzimidazole drugs (albendazole (ALB) and mebendazole (MEB)), which make these campaigns highly vulnerable to the development of anthelminthic resistance [2-5]. Second, these benzimidazole drugs cannot be administered to women in the first trimester of pregnancy due to their teratogenicity [6]. Thirdly, a single dose of the benzimidazole drugs against the different STH is often not sufficient to clear infections completely, showing primarily poor efficacy against *T. trichiura* infections, especially when levels of infection intensity are high [7-10]. Despite the pressing and obvious need for alternative drugs, there are currently no compounds available on the market that outcompete the benzimidazoles in terms of efficacy, safety and feasibility [11-15].

Studies going back many decades have indicated that the cysteine proteinases (CPs) from papaya (*Carica papaya*), collectively known as ‘papain’, have the ability to control gastrointestinal parasites in humans, including STH [16-18]. The CPs of papaya reside in the latex that is present in most parts of the plant (but not the ripe fruit) and is released upon injury. Plant derived CPs are thought to help protect the developing and unripe fruits from exploitation by plant-parasitic nematodes and by arthropod pests [19-21]. It is known that there are four CPs in the latex, of which papain is the least abundant [22]. Confusingly, crude preparations of papaya latex CPs are often referred to as ‘papain’, but we will use the term ‘papaya CPs’. The anthelmintic application of papaya CPs in public health subsequently received very little attention, probably due to competition with the upcoming synthetic anthelmintic drugs. However, it is already apparent that papaya CPs are most likely safe and tolerable, as they are already present in the human food chain (e.g., meat tenderizers, beer clarifiers, and enhancers of food item flavours [23,24]. Second, papaya CPs with a molecular weight of ~23.5 kDa are very unlikely to be absorbed without first being broken down to peptides, and hence can probably be administered to women at an early stage of pregnancy. In addition, papaya CPs have a different mode of action to current anthelmintics (digestion of helminth cuticles) [25], and hence they are a serious contender for an alternative backup drug should anthelminthic resistance occur against the benzimidazoles. They are likely to have more than one target site on the cuticle, making rapid development of resistance very unlikely. Finally, their impact is not restricted to STH, but may have also a clear impact on other gastro-intestinal parasites, as shown by the in vitro experiments on animal tapeworms [25,26].

With increasing reports of resistance against current synthetic anthelminthics in veterinary medicine and lack of efficacy in some cases, the efficacy of papaya CPs has mainly been evaluated against gastrointestinal nematodes in sheep (*Haemonchus contortus*) [27] and also in various in vivo laboratory models [28-32]. In the present study, we evaluated the efficacy of papaya CPs against experimental *T. suis* infections in pigs, the latter being an appropriate and relevant animal model for human trichuriasis. This host-parasite combination allows the assessment of efficacy by means of reduction in both egg counts and adult worm counts. In addition, we verified whether egg counts in stool are an appropriate proxy for worm burden.

## Methods

### Trial design

Two randomized control efficacy trials were conducted. In the first trial, we assessed how a single-oral dose of 450 μmol active papaya CPs compares with a single-oral dose of 400 mg ALB (proof-of-principle trial) for low and high intensity *T. suis* infections. In the second trial, we determined to what extent we could reduce the dose of papaya CPs without loss of the efficacy levels obtained by administering 450 μmol papaya CP (dose–response trial).

In each of these trials, Rattlerow Senghers hybrid piglets between 8–12 weeks were infected with *T. suis* eggs containing infectious larvae at day (D)-42. At D-1, levels of egg excretion by means of fecal egg counts (FECs) were determined, after which animals were randomized to a treatment group stratifying for level of egg excretion. The treatments were given on D0. By this time, the mean body weight of the pigs was 43.3 kg (95% confidence interval (95% CI): 40.2-46.2). Each treatment group included six animals. Finally, at D + 6 FECs were determined again and at D + 7 all the animals were sacrificed and the total number of adult *T. suis* was assessed. Although each of the two trials followed this general format, they differed in the number of *T. suis* (inoculum), the product and the dose of the product administered. In the proof-of-principle trial, we assessed the efficacy of a single-oral dose of 450 μmol papaya CPs and a single-oral dose of 400 mg ALB against low (inoculum = 300 embryonated *T. suis* eggs) and heavy intensity *T. suis* infections (inoculum = 3,000 embryonated *T. suis* eggs). In the dose–response trial, we assessed the efficacy of a single oral-dose of 45 (10% of the dose evaluated in the proof-of-principle trial), 115 (25%), 225 (50%) and 450 μmol (100%) of papaya CPs against high levels of infection intensity (inoculum = 3,000 *T. suis* eggs). The Ethical Committee of the Faculty of Veterinary Medicine, Ghent University (Belgium) approved the study protocol of both trials (Trial 1: EC2011/171; Trial 2 EC2012/178). The animals were bred, raised and housed at the Animal Experimental Unit (*Biocentrum Agrivet*) of the Faculty of Veterinary Medicine, Ghent University, Belgium.

### Papaya cysteine proteinase and albendazole

A supernatant from papaya latex enriched in CP activity was prepared as described [27]. Briefly, spray-dried *C. papaya* latex (Enzymase P1, Enzymase International S.A., 33 rue Jean-Baptiste BAECK - bte 3, B - 1190 Brussels, Belgium) was suspended in water then centrifuged at 17,700 × *g*. The supernatant was taken and concentrated by dialysis over polyethylene glycol 20,000. The concentrated material was aliquotted into individual containers and freeze-dried. Sample aliquots were taken at various time-points, weighed, and the concentration of active CP was determined by active-site titration with the CP inactivator E-64 (L-*trans*-epoxysuccinyl-leucylamido 4-guanidino butane – Sigma Aldrich) [33]. Once aliquotted and freeze-dried, there was no detectable deterioration of enzyme activity over a period of at least 2 years, the specific activity of the material remaining at 10 μmol/g. The 400 mg ALB originated from GlaxoSmithKline Pharmaceuticals Limited, India.

### Inoculation of *T. suis* eggs

For each animal, the inoculum of 300 or 3,000 embryonated *T. suis* eggs was suspended in 5 ml of tap water, and subsequently administered directly into the stomach of the animal by gavage. To avoid accumulation of the *T. suis* eggs, the tube used to gavage was flushed with 10 ml of tap water before inoculating the next animal in the infection sequence. The *T. suis* eggs were kindly provided by Peter Nejsum and Stig Milan Thamsborg (Department of Veterinary Disease Biology, Faculty of Health and Medical Sciences, University of Copenhagen, DK-1870 Frederiksberg C, Denmark).

### Administration of papaya CPs and ALB

Both papaya CPs and ALB tablets were crushed to powder using a pestle and mortar. Subsequently, the powder was suspended in tap water. The volume in which the papaya CPs was suspended ranged from 20 ml (45 μmol) to 150 ml (450 μmol). One ALB tablet was suspended in 20 ml of tap water. Both papaya CPs and ALB were administered directly into the stomach of the animal by gavage. The control group received 30 ml of tap water administered directly into the stomach by gavage. To avoid accumulation of the products, the tube used to gavage was flushed with 10 ml of tap water before inoculating the following animal. In addition to this, a separate tube was used for each treatment group to avoid cross-contamination.

### Parasitological examination

#### Assessment of FEC

FECs were determined using the McMaster egg counting method based on a protocol described by the Ministry of Agriculture, Fisheries, and Food (1986) [34]. Briefly, four grams of stool were suspended in 60 ml of saturated salt solution at room temperature (density ~ 1.2), prepared by adding NaCl to 5 l of warm distilled water (40–50°C) until no more salt went into solution and the excess settled on the bottom of the container). The fecal suspension was poured three times through a wire mesh (aperture of 250 μm) to remove large debris. Then, 0.5 ml aliquots were added to each of the two chambers of a McMaster slide ( http://www.mcmaster.co.za). Both chambers were examined under a light microscope using a 100x magnification and the FECs, expressed as eggs per gram of stool (EPG), were obtained by multiplying the total number of eggs by 50.

#### Assessment of total adult T. suis worm counts

After sacrificing the animals, the large intestine (from caecum to rectum) was removed from the abdominal cavity. Then, the large intestine was opened longitudinally and rinsed with water to remove the content. The content of the intestine was further thoroughly washed on wire mesh (aperture of 800 μm), withholding the adult *T. suis*. Finally, adult *T. suis* worms were counted both in situ in the mucosa of the large intestine and in the contents of the intestine. To obtain the total adult *T. suis* worm counts (WC), the number of adult worms found in the mucosa of the large intestine was added to the number of worms found in the content of the intestine.

### Statistical analysis

Statistical analysis consisted of three parts. First, we verified whether animals were successfully randomized based on FECs. To this end, arithmetic mean FEC at D-1 was summarized for each of the different treatment groups in the two trials (in the first trial this was done for each level of infection intensity separately). Corresponding 95% confidence intervals were determined by bootstrap analysis (10,000 iterations). Differences in FECs at D-1 across the treatment groups were evaluated by a permutation test based on the F-statistic (10,000 iterations). Second, we report the efficacy of the different treatment regimens against *T. suis* infections in each trial by means of egg reduction rate (ERR) at D + 6 and the adult *T. suis* worms at D + 7 reduction rate (WRR) compared to the control group using the formulae below:

$$\begin{array}{ll} \;\text{ERR} & =100\%\\ & \;\times \;\left(1-\frac{\text{arithmetric mean of FECs at}\;D+6\;\text{in the treatment group}}{\text{arithmetric of FECs at}\;D+6\;\text{in the control group}}\right)\\ \;\text{WRR} & =100\%\\ & \;\times \;\left(1‒\frac{\text{arithmetric mean of WCs at}\;D+7\;\text{in the treatment group}}{\text{arithmetric mean of WCs at}\;D+7\;\text{in the control group}}\right) \end{array}$$

Corresponding 95% confidence intervals were determined by bootstrap analysis (10,000 iterations). In the proof-of-principle trial, differences in efficacy between a single-oral dose of CPs and ALB were evaluated for each of the two levels of infection intensity using a permutation test based on the t-statistic (10,000 iterations). For the dose–response trial, any difference in efficacy between the four doses of CPs was evaluated, using a permutation test based on the t-statistic (10,000 iterations). Tukey’s method was applied for multiple comparisons. Third, we evaluated whether FECs are an appropriate proxy for worm burden. To this end, we determined the correlations between the WC at D+7 and the corresponding FECs at D+6, for all animals in both trials by calculating the Pearsons’ correlation coefficient. The statistical analysis was conducted in the statistical software R (version 3.0.1, 2013, The R Foundation for Statistical Computing Platform). The level of significance for each test was set at *p* ≤0.05.

## Results

### Proof-of-principle trial

Table 1 summarizes the number of animals, mean FECs at D-1, the mean FECs at D+6 and the mean WCs at D+7 for each of the three treatment groups (single-oral dose of 450 μmol papaya CPs [CP450], single-oral dose of 400 mg ALB, control), and the efficacy of CP450 and ALB for low and high intensity *T. suis* infections, separately. One animal in the CP450 group inoculated with 3,000 *T. suis* eggs died during the trial. Autopsy did not reveal any causal association between the CP450 treatment and death. In the remaining groups, all 6 animals completed the trial. The mean FECs at D-1 for animals inoculated with 300 eggs, ranged from 8.3 EPG (95% CI: 0.0; 25.0) in both the P450 and ALB group to 16.7 EPG (95% CI: 0.0; 50.0) in the control group. For animals inoculated with 3,000 eggs, the mean FEC at D-1 ranged from 366.7 EPG (95% CI: 58.3; 800.0) in the control group to 483.3 (95% CI: 116.7; 916.7) in the ALB. For both levels of infection intensity, there was no significant difference in FEC at D-1 across the different treatment groups (F_low_ = 0.17, *p* = 0.78, F_high_ = 0.08, *p* = 0.93). The mean WC at D+7 in the control animals inoculated with 300 eggs was 131.5 (95% CI: 107.2; 161.3, recovery rate of 43.8% [95% CI: 35.7; 53.8]), for the control animals inoculated with 3,000 eggs the mean WC equaled 1,142.2 (95% CI: 893.0; 1,419.0, recovery of 38.1% [95% CI: 29.8; 47.3]). The ratio of mean FECs at D+6 and WCs at D+7 in the control animals inoculated with 3,000 eggs and the control animals inoculated with 300 eggs was 18.6 [95% CI: 5.2; 57.0] and 8.7 [95% CI: 6.3; 11.8], respectively.

**Table 1:** **The efficacy of papaya CPs and albendazole against low and high intensity *T. suis *** **infections**

	**n**	**Mean FECs at D-1 (EPG) (95% CI)**	**Mean FECs at D+6 (EPG) (96% CI)**	**ERR (%) (95% CI)**	**Mean WCs at D7 (95% CI)**	**WRR (%) (95% CI)**
*Low infection intensity (inoculum = 300 eggs)*						
CP450	6	8.3(0.0; 25.0)	8.3(0.0; 25.0)	98.3(92.7; 100)	1.3(0.2; 2.8)	99.0*(97.7; 99.9)
ALB	6	8.3(0.0; 25.0)	175.0(16.7; 358.3)	64.4(-15.8; 96.3)	80.2(40.8; 122.0)	39.0*(2.4; 69.1)
Control	6	16.7(0; 50.0)	491.7(183.3; 800.0)	_	131.5(107.2; 161.3)	_
*High infection intensity (inoculum = 3,000 eggs)*						
CP450	5**	420.0(60.0; 780.0)	100.0(10.0; 230.0)	98.9*(95.5; 99.9)	29.2(3.2; 63.2)	97.4*(94.3; 99.7)
ALB	6	483.3(116.7; 916.7)	3800.0(1908.3; 5666.7)	59.0*(-47.0; 83.4)	877.2(597.0; 1,094.3)	23.2*(-7.8; 49.7)
Control	6	366.7(58.3; 800.0)	9266.7(2683.3; 17416.7)	_	1142.2(893.0; 1,419.0)	_

The fecal egg counts (FEC) at day (D) -1, mean worm counts (WC) at D+7 and the reduction in WC at D+7 compared to the control group (WCR).

*pair-wise comparison between CP450 and ALB revealed a significant difference with *p*-value <0.01; **One animal which received 3,000 eggs of *T. suis* and which was assigned to the CP450 treatment died in the course of trial. Autopsy did not reveal any causal association between the treatment and death.

A single-oral dose of P450 was highly efficacious, resulting in a ERR and WRR of almost 100% for both low (ERR: 98.3 [95% CI: 92.7; 100]; WRR: 99.0% [95% CI: 97.7; 99.9]) and high intensity infections (ERR: 98.9 [95% CI: 95.5; 99.9]; WRR: 97.4%% [95% CI: 94.3; 99.7]). A single-oral dose of ALB showed poor efficacy against both levels of infection intensity (low infection intensity: ERR: 64.4% [95% CI: -15.8; 96.3]; WRR: 39.0% [95% CI: 2.4; 69.1], high infection intensity: ERR: 59.0% [95% CI: -47.0; 83.4]; WRR: 23.2% [95% CI: -7.8; 49.7]). For both levels of infection intensity, a single-oral dose of P450 was significantly more efficacious compared to a single-oral dose of ALB by means of WRR (t_low_ = 3.5, *p* = 0.003; t_high_ = 6.1, *p* = 0.005). When the efficacy was assessed by means of ERR, a significant difference was only observed for high levels of infection intensity, CP450 being more efficacious than ALB (t = 3.5, *p* = 0.006). For low level infection intensity, there was no significant difference in ERR between CP450 and ALB (t = 1.71, *p* = 0.15).

### Dose–response trial

Table 2 summarizes the number of animals, mean FEC at D-1, the mean FEC D+6, and WC at D7 for each of the five treatment groups (single-oral dose of 45, 115, 225 and 450 μmol CP [CP45, CP115, CP225 and CP450] and control) against high-intensity *T. suis* infections. All 30 animals (6 animals per treatment group) completed the trial. The mean FEC at D-1 ranged from 233.3 EPG (95% CI: 16.7; 558.3) for the CP115 group to 500.0 EPG (95% CI: 41.7; 1,200.0) for the CP45 group. There was no significant difference in FEC at D-1 across the different treatment groups (F = 0.219, *p* = 0.95). The mean WC at D+7 in the control animals was 856.3 (95%CI: 328.7; 1,384.0, recovery rate of 28.5% [95% CI: 11.0; 46.1]).

**Table 2:** **The efficacy of four doses of papaya CPs against high intensity *T. suis *** **infections**

**Treatment arm**	**n**	**Mean FEC at D-1 (EPG) (95% CI)**	**Mean FEC at D+6(EPG) (95% CI)**	**ERR (%) (95% CI)**	**Mean WC at D+7 (95% CI)**	**WRR (%) (95% CI)**
CP45	6	500.0(41.7; 1,200)	5416.7(2200.0; 9233.3)	2.1*(-629.8; 68.9)	976.5(697.3; 1,256.1)	-14.0*(-213.7; 36.1)
CP115	6	233.3(16.7; 558.3)	1425.0(183.3; 2908.3)	74.2(-98.1; 96.9)	278.5(64.7; 551.7)	67.5(-3.4; 93.9)
CP225	6	241.7(50.0; 533.5)	808.3(208.3; 1741.7)	85.4(-15.4; 97.1)	160.3(61.3; 265.8)	81.3(43.7; 93.9)
CP450	6	441.7(25.0; 1,150.0)	41.7(0.0; 108.3)	99.2*(93.6; 100)	8.5(2.7; 15.7)	99.0*(97.0; 99.7)
Control	6	308.3(33.3; 733.3)	5533.3(758.3; 11608.3)	_	856.3(328.7; 1,384.0)	_

The mean fecal egg counts (FEC) at day (D) -1, mean worm counts (WC) at D+7 and the reduction in WC at D+7 compared to the control group (WCR) for a single-oral dose of 45, 115, 225, and 450 μmol papaya CPs (P45, P115, P225 and P450).

*pair-wise comparison between CP450 and CP45 revealed a significant difference with *p*-value <0.01.

**Figure 1:**
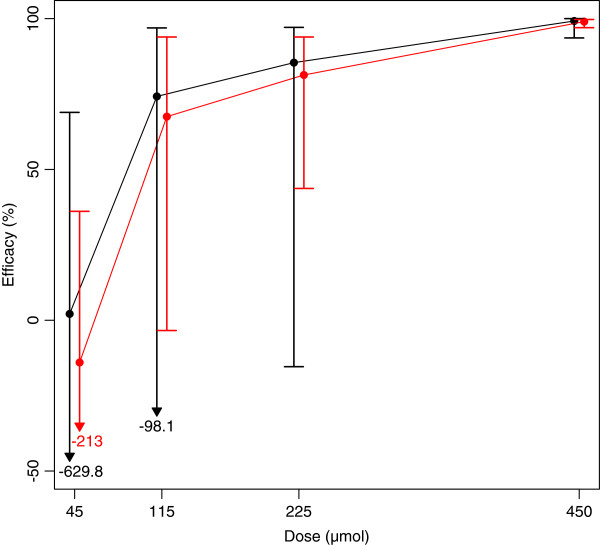
**The efficacy of four doses of papaya CPs against high intensity *T. suis *infections assessed by means of egg reduction rate (black) and worm reduction rate (red).** Points represent mean values (n=6) and error bars show 95% CI with inserted values where bars extend beyond the Y-axis.

Overall, the efficacy measured by ERR and WRR increased as a function of increasing dose of CP. For ERR, the results ranged from 2.1% (95% CI: -629.8; 68.9) for a single oral dose of CP45 to 99.2% (95% CI: 93.6; 100) for a single oral dose of CP450. For WRR these results ranged from -14.0% (95% CI: -213.7; 36.1) to 99.0% (95% CI: 97.0; 99.7), respectively. As illustrated in Figure 1, there was initially a relatively small fall in efficacy as the dose was reduced from CP450 to CP115, and then a steep drop in efficacy towards CP45. A single-oral dose CP450 was more efficacious compared to a single-oral dose of CP45 by means of both ERR (t = 2.7, *p* = 0.005) and WRR (t = 6.1, *p* = 0.001). The remaining pair-wise comparisons did not reveal a significant difference in efficacy (*p* >0.12).

### FEC as a proxy for worm burden

**Figure 2:**
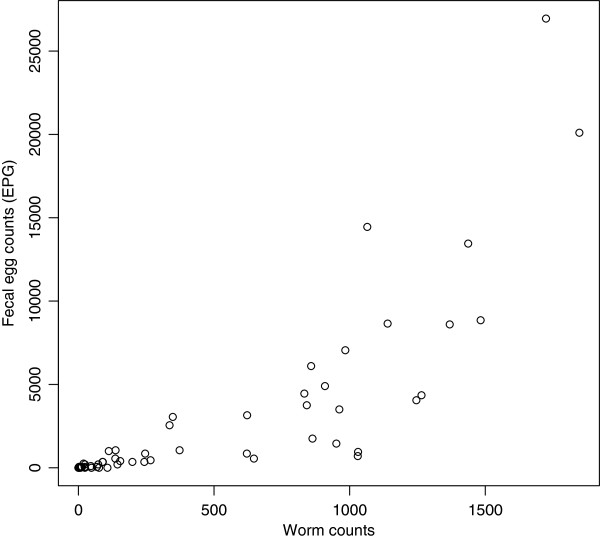
Scatterplots describing the fecal egg counts as a function of the worm counts.

Overall, there was high correlation (Rs = 0.80, *p* <0.0001) between WC and FEC. However, as illustrated in Figure 2, the relationship between these two variables was not linear, rather the FECs increased as an exponential function of WCs (R^2^ = 0.83).

## Discussion

In the present study we conducted two randomized controlled efficacy trials to evaluate the efficacy of papaya CPs against experimental *T. suis* infections in pigs, and we clearly demonstrated that a single application of CPs at the higher doses used for treatment is far more effective than the most widely used synthetic anthelmintic. Therefore, this study confirms that papaya CPs have anthelminthic properties that are extremely effective against *Trichuris* infections. Here a single-oral dose of 450 μmol of papaya CPs reduced the egg excretion and worm burden by more than 97%, regardless of the level of infection intensity. These efficacy results are superior not only to the efficacy results of a single-oral dose of 400 mg ALB in the current study, but also to most ERR efficacy results reported in human populations for ALB and MEB, either administered in a single oral dose on one day (1×1 ALB: 64.5%; 1×1 MEB: 62.7%, Levecke et al., unpublished data), or even in several doses over consecutive days (2×1 ALB: 73.5%, 2×1 MEB: 87.1% [35], 3×1 ALB: 94.0%, 3×1 MEB: 97.3% [36], a double oral dose on 1 day (1×2 ALB: 94.8%, 1×2 MEB: 90.3% [37]. Single dose CP treatment was also considerably more effective in removing *T. suis* than any of the other compounds trialed in recent years (1× levamisol: 0% [38], 1× ivermectin: 86.8% [38], 1× tribendimidine: 31.1% [11], 1× nitazoxanide: 13.4% [12]) and drug combinations (1×1 ALB+MEB: 96.1%; 2×1 ALB+MEB: 97.3% [37], 1×1 ALB+ivermectin: 91.1% [38] and 97.5% [39]; ALB+diethylcarbamazine: 79.4% [39]; 2×1 ALB+nitazoxanide: 54.9% [35]; 1×1 MEB+levamisole: 85.0% [40]; 1×1 MEB+ivermectine: 96.7% [38]; pyrantel-oxantel: 86.9% [41].

Despite these promising results, there are some aspects that still need to be addressed before papaya CP can be considered as an acceptable alternative anthelminthic drug for MDA programs in the control of STH. These include formulation, dose and safety. At present, a dose 450 μmol papaya CPs represents a mass of ~45 g, and this poses an important obstacle in treating both human patients and animals but particularly a pediatric population due to the quantity of material. Nevertheless, given that the CPs used in the current work were contained entirely in a solution (unlike in some earlier work where gel-like latex was used, Stepek et al. [21], further concentration is possible, and a range of different vehicles/matrices can be exploited to generate a formulation and delivery system in a smaller volume that is more acceptable for oral delivery.

The dose–response trial indicated that the dose can be halved (225 μmol), without losing too much of its efficacy (~99% vs. ~80%). Although this reduction in dose will also half the mass of CP, it still remains substantial (~22 g) and in terms of weight and bulk, more than alternative, albeit less effective, treatments.

‘Papain’ has found various applications in the daily life of people and in the food industry, and is therefore widely available for these purposes, with less regulation than conventional medicines. However, the amounts of papain used by the consumer for these tasks are clearly much lower than the minimal dose that showed anthelmintic efficacy against trichuriasis in our trials (115 μmol or ~11 g). Some side effects of papaya CPs have been reported previously in animals and humans but these are relatively scarce. In the sheep trials reported by Buttle et al. [27], some oral blistering was observed, but this was not serious at the doses used [27]. Regurgitation of CP and their subsequent inhalation present a more serious threat, but again when administered to animals by well-trained staff oral delivery to mice did not result in losses. Novel formulations based on syrups would also most-likely eliminate this as a problem in treatment of people. CPs are proteins and there is also the risk of allergy developing to their frequent application. However, it should be emphasized here that the side effects have only been evaluated poorly to date and that this is one aspect of the use of papaya CPs that will have to be more thoroughly investigated before they can be marketed as anthelmintics.

The poor efficacy of ALB against *T. suis* (ERR: 59.0-64.4; WRR: 23.2-39.0) was not entirely unexpected, as the ERR results resemble those reported in a recent meta-analysis including five trials assessing the efficacy of a single oral-dose of 400 mg ALB in school children Cameroon, Ethiopia, Tanzania and Vietnam (ERR: 64.5% [95% CI: 44.4; 84.7]; Levecke et al., unpublished data). Although our results confirm the usefulness of pigs as a model for assessing drug efficacy against trichuriasis, we expected a more pronounced difference in efficacy across the two levels of infection intensity. The ERR for ALB against trichuriasis in the five aforementioned trials with school children ranged from 29.3% for a mean FEC at baseline of 1193 EPG, to 92.4% for a mean FEC at baseline of 420 EPG. In the current study, ALB provided comparable ERR results against low (mean FEC = 492 EPG, ERR = 64.4%) and heavy-intensity infections (mean FEC = 9267 EPG, ERR = 59.0%). Only for WRR was there a pronounced difference in efficacy (low: 23.2% vs. heavy: 39.0%).

Finally, our results indicate that egg excretion increased as a function of worm burden, suggesting that FEC are a valid proxy of worm burden for trichuriasis in pigs.

## Conclusions

A single dose of 450 μmol papaya CPs provided greater efficacy against *T. suis* infections in pigs than a single-oral dose of 400 mg ALB. Although these results highlight the possibility of papaya CPs being used to treat human trichuriasis further development is needed in order to obtain and validate an oral formulation for use in both human and veterinary medicine. Our results confirm the suitability of pigs as a model for assessing drug efficacy against trichuriasis. Finally, this study suggests that FEC are a valid proxy for worm burdens in this host-parasite system.

## Abbreviations

CP: Cysteine proteinase; STH: Soil-transmitted helminths; ERR: Egg reduction rate; WRR: Worm reduction rate; ALB: Albendazole; NTD: Neglected tropical diseases; MDA: mass drug administration; MEB: Mebendazole; D: Day; FEC: Fecal egg count; WC: Worm count.

## Competing interests

The authors declare that they and their institutions have no financial or personal relationships with other people or organizations that could inappropriately influence (bias) their actions.

## Authors’ contributions

BL: conception and design of the trials; statistical analysis and interpretation of the data, conducting the trials, writing up the manuscript; DJB: conception and design of the trials; preparation and standardization of papaya CPs, interpretation of the data, writing up the manuscript. JMB and IRD: conception and design of the trials, interpretation of the data, writing up the manuscript. JV: conception and design of the trials, interpretation of the data, writing up the manuscript. All authors read and approved the final version of the manuscript.

## References

[B1] NTD Partner Website [ http://www.unitingtocombatntds.org]

[B2] Geerts S Gryseels B Drug resistance in human helminths: current situation and lessons from livestock Clin Microbiol Rev 2000 13 202 222 10.1128/cmr.13.2.207-222.2000 PMC100151 10755998

[B3] Vercruysse J Albonico M Behnke JM Kotze AC Prichard RK McCarthy JS Montresor A Levecke B Is anthelmintic resistance a concern for the control of human soil-transmitted helminths? Int J Parasitol Drugs and Drug Resistance 2011 1 14 27 10.1016/j.ijpddr.2011.09.002 PMC3913213 24533260

[B4] Humphries D Mosites E Otchere J Twum WA Woo L Jones-Sanpei H Harrison LM Bungiro RD Benham-Pyle B Bimi L Edoh D Bosompem K Wilson M Cappello M Epidemiology of hookworm infection in Kintampo North Municipality, Ghana: patterns of malaria coinfection, anemia, and albendazole treatment failure Am J Trop Med Hyg 2011 84 792 800 10.4269/ajtmh.2011.11-0003 21540391 PMC3083749

[B5] Soukhathammavong PA Sayasone S Phongluxa K Xayaseng V Utzinger J Vounatsou P Hatz C Akkhavong K Keiser J Odermatt P Low efficacy of single-dose albendazole and mebendazole against hookworm and effect on concomitant helminth infection in Lao PDR PLoS Negl Trop Dis 2012 6 e1417 10.1371/journal.pntd.0001417 22235353 PMC3250499

[B6] Dayan AD Albendazole, mebendazole and praziquantel. Review of non-clinical toxicity and pharmacokinetics Acta Trop 2003 86 141 159 10.1016/S0001-706X(03)00031-7 12745134

[B7] Bennett A Guyatt H Reducing intestinal nematode infection: efficacy of albendazole and mebendazole Parasitol Today 2000 16 71 74 10.1016/S0169-4758(99)01544-6 10652492

[B8] Keiser J Utzinger J Efficacy of current drugs against soil-transmitted helminth infections - systematic review and meta-analysis JAMA 2008 299 1937 1948 18430913 10.1001/jama.299.16.1937

[B9] Vercruysse J Behnke JM Albonico M Ame SM Angebault C Bethony JM Engels D Guillard B Hoa NTV Kang G Kattula D Kotze AC McCarthy JS Mekonnen Z Montresor A Periago MV Sumo L Tchuem Tchuenté L-A Thach DTC Zeynudin A Levecke B Assessment of the anthelmintic efficacy of albendazole in school children in seven countries where soil-transmitted helminths are endemic PLoS Negl Trop Dis 2011 5 e948 10.1371/journal.pntd.0000948 21468309 PMC3066140

[B10] Levecke B Mekonnen Z Albonico M Vercruysse J The impact of baseline FEC on the efficacy of a single-dose albendazole against *Trichuris trichiura* Trans R Soc Trop Med Hyg 2012 106 128 130 10.1016/j.trstmh.2011.09.007 22189084

[B11] Steinmann P Zhou XN Du ZW Jiang JY Xiao SH Wu ZX Zhou H Utzinger J Tribendimidine and albendazole for treating soil-transmitted helminths, *Strongyloides stercoralis* and *Taenia* spp.: open-label randomized trial PLoS Negl Trop Dis 2008 2 e322 10.1371/journal.pntd.0000322 18923706 PMC2561005

[B12] Olliaro P Seiler J Kuesel A Horton J Clark JN Don R Keiser J Potential drug development candidates for human soil-transmitted helminthiases PLoS Negl Trop Dis 2011 5 e1138 10.1371/journal.pntd.0001138 21695247 PMC3111745

[B13] Tritten L Silbereisen A Keiser J In vitro and in vivo efficacy of monepantel (AAD 1566) against laboratory models of human intestinal nematode infections PLoS Negl Trop Dis 2011 5 e1457 10.1371/journal.pntd.0001457 22216366 PMC3246443

[B14] Speich B Ame SM Ali SM Alles R Hattendorf J Utzinger J Albonico M Keiser J Efficacy and safety of nitazoxanide, albendazole, and nitazoxanide-albendazole against *Trichuris trichiura* infection: a randomized controlled trial PLoS Negl Trop Dis 2012 6 e1685 10.1371/journal.pntd.0001685 22679525 PMC3367984

[B15] Keiser J Tritten L Silbereisen A Speich B Adelfio R Vargas M Activity of oxantel pamoate monotherapy and combination chemotherapy against *Trichuris muris* and hookworms: revival of an old drug PLoS Negl Trop Dis 2013 7 e2119 10.1371/journal.pntd.0002119 23556013 PMC3605275

[B16] Berger J Asenjo CF Anthelmintic activity of crystalline papain Science 1940 91 387 388 10.1126/science.91.2364.387 17773432

[B17] Jonxis JHP Bekius H Treatment of *Ascaris* infection with Velardon Arch Dis Child 1953 140 329 331 13081173 10.1136/adc.28.140.329 PMC1988707

[B18] Stransky E Reyes A Ascariasis in the tropics (with considerations on its treatment) J Trop Ped 1955 1 174 187 10.1093/oxfordjournals.tropej.a057363 24543887

[B19] Konno K Hirayama C Nakamura M Tateishi K Tamura Y Hattori M Kohno K Papain protects papaya tress from herbivorous insects: role of cysteine proteases in latex Plant J 2004 37 370 378 10.1046/j.1365-313X.2003.01968.x 14731257

[B20] Miller PM Sands DC Effects of hydrolytic enzymes on plant parasitic nematodes J Nematol 1977 9 192 197 19305592 PMC2620239

[B21] Stepek G Curtis RHC Kerry BR Shewry PR Clark SJ Lowe AE Duce IR Buttle DJ Behnke JM Nematicidal effects of cysteine proteinases against sedentary plant parasitic nematodes Parasitology 2007 134 1831 183 17640402 10.1017/S0031182007003289

[B22] Buttle DJ Dando PM Coe PF Sharp SL Shepherd ST Barret AJ The preparation of fully active chymopapain free of contaminating proteinases Biol Chem Hoppe-Seyler 1990 371 1083 1088 10.1515/bchm3.1990.371.2.1083 2085414

[B23] Caygill JC Sulphydryl plant proteases Enzyme Microb Technol 1979 1 233 242 10.1016/0141-0229(79)90042-5

[B24] Pritchard EI Pre-slaughter treatment in relation to nutritional quality of meat Proc Inst Food Sci Technol 1971 4 118 123

[B25] Behnke JM Buttle DJ Stepek G Lowe A Duce IR Developing novel anthelmintics from plant cysteine proteinases Parasit Vectors 2008 1 29 10.1186/1756-3305-1-29 18761736 PMC2559997

[B26] Mansur F Luoga W Buttle DJ Duce IR Lowe A Behnke JM The anthelmintic efficacy of natural plant cysteine proteinases against two rodent cestodes *Hymenolepis diminuta* and *Hymenolepis microstoma* in vitro Vet Parasitolin press 10.1016/j.vetpar.2013.12.018 24462509

[B27] Buttle DJ Behnke JM Bartley Y Elsheikha HM Bartley DJ Garnett MC Donnan AA Jackson F Lowe A Duce IR Oral dosing with papaya latex is an effective anthelmintic treatment for sheep infected with *Haemonchus contortus* Parasit Vectors 2011 4 36 10.1186/1756-3305-4-36 21406090 PMC3068120

[B28] Stepek G Lowe AE Buttle DJ Duce IR Behnke JM In vitro and in vivo anthelmintic efficacy of plant cysteine proteinases against the rodent gastrointestinal nematode, *Trichuris muris* Parasitology 2006 132 681 689 16448585 10.1017/S003118200500973X

[B29] Stepek G Lowe AE Buttle DJ Duce IR Behnke JM The anthelmintic efficacy of plant-derived cysteine proteinases against the rodent gastrointestinal nematode, *Heligmosomoides polygyrus*, in vivo Parasitology 2007 134 1409 1419 10.1017/S0031182007002867 17475089

[B30] Satrija F Nansen P Murtini S He S Anthelmintic activity of papaya latex against patent *Heligmosomoides polygyrus* infections in mice J Ethnopharmacol 1995 48 161 164 10.1016/0378-8741(95)01298-R 8719976

[B31] Stepek G Lowe AE Buttle DJ Duce IR Behnke JM Anthelmintic action of plant cysteine proteinases against the rodent stomach nematode, *Protospirura muricola*, in vitro and in vivo Parasitology 2007 134 103 112 10.1017/S0031182006001302 17032468

[B32] Luoga W Mansur F Buttle DJ Duce IR Garnett MC Lowe A Behnke JM The relative anthelmintic efficacy of plant-derived cysteine proteinases on intestinal nematodes J Helmintholin press 10.1017/S0022149X13000692 24176056

[B33] Zucker S Buttle DJ Nicklin MJH Barrett AJ Proteolytic activities of papain, chymopapain and papaya proteinase III Biochim Biophys Acta 1985 828 196 204 10.1016/0167-4838(85)90057-3 3919769

[B34] Ministry of Agriculture Fisheries and Food: Manual of Veterinary Parasitological Laboratory Techniques 1986 London: Her Majesty’s Stationery Office

[B35] Mekonnen Z Levecke B Boulet G Bogers JP Vercruysse J Efficacy of different albendazole and mebendazole regimens against high-intensity *Trichuris trichiura* infections in school children, Jimma, Ethiopia Pathog Glob Health 2013 107 207 209 10.1179/2047773213Y.0000000092 23816513 PMC4001473

[B36] Steinmann P Utzinger J Du ZW Jiang JY Chen JX Hattendorf J Zhou H Zhou XN Efficacy of single-dose and triple-dose albendazole and mebendazole against soil-transmitted helminths and *Taenia* spp.: a randomized controlled trial PLoS One 2011 6 e25003 10.1371/journal.pone.0025003 21980373 PMC3181256

[B37] Namwanje H Kabatereine NB Olsen A Efficacy of single and double doses of albendazole and mebendazole alone and in combination in the treatment of *Trichuris trichiura* in school-age children in Uganda Trans R Soc Trop Med Hyg 2011 105 586 590 10.1016/j.trstmh.2011.07.009 21885077

[B38] Knopp S Mohammed KA Speich B Hattendorf J Khamis IS Khamis AN Stothard JR Rollinson D Marti H Utzinger J Albendazole and mebendazole administered alone or in combination with ivermectin against *Trichuris trichiura*: a randomized controlled trial Clin Infect Dis 2010 51 1420 1428 10.1086/657310 21062129

[B39] Belizario VY Amarillo ME de Leon WU de los Reyes AE Bugayong MG Macatangay BJC A comparison of the efficacy of single doses of albendazole, ivermectin, and diethylcarbamazine alone or in combinations against *Ascaris* and *Trichuris* spp Bull World Health Organ 2003 81 35 42 12640474 PMC2572315

[B40] Albonico M Bickle Q Ramsan M Montresor A Savioli L Taylor M Efficacy of mebendazole and levamisole alone or in combination against intestinal nematode infections after repeated targeted mebendazole treatment in Zanzibar Bull World Health Organ 2003 81 343 352 12856052 PMC2572452

[B41] Albonico M Bickle Q Haji HJ Ramsan M Khatib KJ Montresor A Savioli L Taylor M Evaluation of the efficacy of pyrantel-oxantel for the treatment of soil-transmitted nematode infections Trans R Soc Trop Med Hyg 2002 96 685 690 10.1016/S0035-9203(02)90352-4 12625151 PMC5679355

